# New insights into name category-related effects: is the Age of Acquisition a possible factor?

**DOI:** 10.1186/1744-9081-5-33

**Published:** 2009-07-29

**Authors:** Roberta Adorni, Alice Mado Proverbio

**Affiliations:** 1 Department of Psychology, University of Milano-Bicocca, Viale dell'Innovazione 11, 20125, Milan, Italy

## Abstract

**Background:**

Electrophysiological, hemodynamic and neuropsychological studies have provided evidence of dissociation in the way words belonging to different semantic categories (e.g., animals, tools, actions) are represented in the brain. The aim of the present study was to investigate whether a word's semantic domain may affect the amplitude and latency of ERP components, independently of any other factor.

**Methods:**

EEGs were recorded from 16 volunteers engaged in a lexical decision task (word/non-word discrimination) involving 100 words (flora and fauna names). This task allowed us to evaluate differences in processing between words belonging to different categories (fauna vs. flora) independently of task demands. All stimuli were balanced in terms of length, frequency of occurrence, familiarity and imageability. Low Resolution Electromagnetic Tomography (LORETA) was performed on ERP difference waves of interest.

**Results:**

Our findings showed that the two categories were discriminated as early as 200 ms post-stimulus, with larger responses to flora names over the left occipito-temporal areas, namely BA37 and BA20. Category-related ERP differences were also observed in the amplitudes of the later centro-parietal N400, posterior P300 and anterior LP components. Behavioral responses to words denoting fauna were more accurate than to words denoting flora.

**Conclusion:**

Overall, it seems that it was easier to access the lexical properties of fauna, probably because of their biologically relevant status. The results are discussed in the light of the possible role played by different factors.

## Background

Word comprehension requires access to semantic knowledge. How concepts are organized in the brain is a central theme of cognitive neuroscience in which at least two classes of debates interweave: the first about the existence of a single amodal semantic system vs. multiple semantic systems; the second about the existence of specific semantic category neural circuits, rather than cerebral activations emerging from the distinctive features of the various concepts, such as the class of knowledge (perceptual vs. functional/associative) or the modality (visual vs. verbal).

In this context, different theoretical accounts have been proposed. For instance, the Sensory/Functional Theory [1,2] states that the semantic system is organized into modality-specific subsystems. Given that visual attributes are more relevant to representing natural entities, whereas action-related attributes are more relevant to representing artificial objects, this assumes that the ability to recognize/name living things depends on visual/perceptual information, whereas the ability to recognize/name non-living things depends on functional/associative information. Another account, the Organized Unitary Content Hypothesis – the OUCH model [3,4] – assumes that conceptual knowledge corresponding to object properties that co-occur with high frequency is stored contiguously in semantic space. For instance, a focal cerebral lesion can cause category-specific semantic deficits either because conceptual knowledge corresponding to things with similar features is stored in nearby neural structures or because damage to a given property propagates the deficit to highly correlated properties. The Domain-Specific hypothesis [5] underlines the role of evolutionary pressures, which may have resulted in specialization as well as functional dissociation of the neural circuits responsible for the perceptual and conceptual processing of different object-categories, in particular the processing of things of which rapid and efficient recognition could entail survival and reproductive advantages. Eligible candidate categories are animals, fruit/vegetables, conspecifics and tools. A final model, the Conceptual-Structure account [6], postulates that semantic categories and domains are not explicitly represented, and categorical dissociations would depend on the specific patterns of brain activation in response to stimuli characterized by specific perceptual and functional features. This model assumes that living entities (most typically animals) have many shared properties (e.g. all mammals breathe, have eyes, can see and eat) that co-occur frequently and are therefore strongly correlated. In contrast, artifacts have fewer properties, which tend to be more distinctive than those of living entities. The conceptual structure account also incorporates the claim that specific perceptual properties become correlated with specific functions. The nature of these form-function correlations distinguishes living from non-living things: artifacts have distinctive forms that are consistently associated with their functions, whereas for living entities, biological function information is highly correlated with shared perceptual properties (e.g. eyes-see).

The scenario depicted by these cognitive models is quite complex and it is difficult to find definitive evidence in favor of one of them. Lesion data have been reported in favor or disfavor of each of the aforementioned models. In our opinion, a promising approach comes from cognitive neuroscience research. For example, many functional neuroimaging studies on lexico-semantic and conceptual processing in healthy volunteers have reported evidence supporting the existence of specific neural circuits for different semantic categories. Most neuroimaging studies examining category-specific differences have used word retrieval tasks (e.g. verbal fluency, picture naming). For instance, in a PET study in which participants were instructed to name pictures of animals, tools and celebrity faces, Damasio and co-workers [7] found that naming celebrity faces activated the left ventro-lateral temporal lobe, whereas naming animals and tools activated different regions of the left inferior posterior temporal cortex, as well as the left temporal pole, suggesting that lexical retrieval of words belonging to different categories may activate different cerebral representations. Generally speaking, functional brain imaging studies have consistently identified the temporal lobe, particularly the posterior region of the left temporal lobe, as a critical site for stored representations, especially of concrete object names. Similar patterns of semantic category-related activity in the ventral and lateral regions of the posterior temporal cortex have now been observed using a range of stimuli. For instance, enhanced activity in the lateral region of the fusiform gyrus (including the Fusiform Face Area) has been found using naming, basic level categorization, or semantic decision tasks with animal pictures and/or their written names. Enhanced activity in the medial fusiform gyrus has been found using tool pictures and/or their written names.

In a recent paper [8], it was emphasized that semantic category effects have mainly been observed during semantic decision and word retrieval tasks in functional neuroimaging studies, but not during tasks that do not require explicit processing of different semantic categories [see also the meta-analysis of Devlin and co-workers, [9]. This aspect challenges the validity of these results, since the meaning of the stimuli is generally accessed even when they are neither attended nor consciously perceived. If it is true that brain activity related to semantic categories mirrors semantic memory organization, then semantic category effects should also emerge during tasks that require no explicit semantic categorization process. In an ERP study using a lexical decision task, Kiefer [8] found a difference in activation in response to words referring to living vs. non-living entities. Living entities were associated with greater positivity in the time windows of the N400 and LP components over the occipito-temporal sites, whereas non-living entities were associated with greater positivity over the fronto-central sites in the time window of N400. He interpreted these results in favor of the existence of multiple semantic systems.

The N400 component is well known to reflect word processing and semantic context integration [10,11]. A few electrophysiological studies have also revealed semantic category effects earlier than the N400 time window, namely in the range of the so-called Recognition Potential or RP [12]. The relatively early latency of the semantic effects should not be surprising considering that recent ERP studies on single word processing have confirmed the existence of lexical effects earlier than the N400 time window [13]. For example, Sereno and collaborators [14] reported a differential response to words vs. pseudo-words as early as 112 ms post-stimulus. Assadollahi and Pulvermüller [15] found early effects of word frequency at about 120–170 ms for short monosyllabic words, and later effects of word frequency (240–270 ms) irrespective of word length. Penolazzi and co-workers [16] found early lexical effects, modulated by word length, as early as 120 ms. Recognition Potential consists of a negative component peaking around 200–250 ms, centered over posterior sites. It has the greatest amplitude in response to words, intermediate in response to legal pseudo-words and lowest in response to illegal strings of letters [17]. In a study focused on investigating the topographical distribution and source localization of the RP, Martín-Loeches and collaborators [18] found different amplitudes of this component in response to animal vs. non-animal names, and by means of BESA they located the possible intracortical RP generator in the basal extrastriate areas, in particular in the lingual and fusiform gyrus. Participants were instructed to press a button in response to animal names (semantic judgment task). Despite the innovative and remarkable results, a potential problem of this study might be that all words belonging to the animal category were target stimuli, whereas all words belonging to the non-animal category were non-target stimuli, so the possibility that the effects observed were somehow contaminated by the attentive selection of targets cannot be excluded, given that the RP is characterized by a latency and topographical distribution similar to the ERP component called Selection Negativity in selective attention paradigms. Indeed, it is known that target stimuli elicit larger Selection Negativity than non-target stimuli [19]. This phenomenon has been described for several visual features, and recently it has been shown in the context of word reading. In another ERP study, Marí-Beffa and co-workers [20] used a priming paradigm presenting pairs of words (prime-probe) about which participants were instructed to solve different tasks. The main purpose of the study was to investigate the role of the task in word semantic processing. Participants had to make an orthographic judgment (search the letter E or A) or a semantic judgment (living vs. non-living) on the prime of each pair, whereas they had to make a lexical decision (word vs. non-word) about the probe. Analysis of the primes showed an early posterior component, identified by the authors as an RP, sensitive to the semantic category of the word but not to the type of processing requested (orthographic vs. semantic). The authors concluded that this sort of initial semantic access was automatic, considering that the later P300 component was affected by task demands as well as by word semantic category. In a recent MEG study [21], using a task in which the participants had to respond only to nouns depicting artificial things (which served as distractors), Assadollahi and Rockstroh found an effect of semantic category (flora names vs. fauna names) as early as 100–150 ms post-stimulus in the left occipito-temporal cortex. The authors interpreted their findings by supposing that the different activations in response to flora and fauna stimuli could not simply be due to visual word form processing, and lexical and semantic effects may occur in different brain regions quite simultaneously. Despite the intriguing results of these two studies, some skepticism about the semantic nature of the effects observed might emerge, considering that the stimuli used might differ in a number of non-balanced aspects, which might have played some role in the cerebral activity. One of the possible confounding effects could be produced by word imageability. Indeed, many studies have shown a focus of activity along the ventral visual pathway during the retrieval of object visual attributes and during explicit imagery tasks [22,23]. In a recent fMRI study, Hauk and collaborators [24] investigated the differential impact of the frequency of occurrence of action-related or highly imaginable words. Stimuli were flashed on the screen for 100 ms and participants had to perform a silent reading task. The authors found a correlation between the frequency of occurrence of the words characterized by high imageability and activation of the bilateral fusiform gyrus.

The purposes of the present study were manifold. First of all, we aimed to investigate word semantic category effects while ruling out the possible confounding effects of some important variables. Moreover, we aimed to control the possible effects of the many features that differentiate man-made things from natural categories of things. For this reason, besides balancing word imageability, familiarity, and other linguistic parameters, we selected two quite "close" semantic categories, usually clustered together under the label "living entities": fauna names and flora names, as previously done by Assadollahi and Rockstroh [21]. Specifically, since flora and fauna names were matched for length, frequency of occurrence, imageability and familiarity, we expected that any difference between the two categories might be interpreted as semantic in nature. Secondly, we aimed to explore cerebral activity in a task that required neither semantic categorization nor explicit semantic information processing. We tried to avoid effects on the kind of processing implemented by the nature of the task, "favoring" one category or the other, or inducing retrieval of a type of information (for example, visual rather than functional) depending on the task resolution strategies put into action. Thus, the purpose was to interfere as little as possible with either the stimulus analysis made by the volunteers or the type of knowledge activated. We predicted that ERP semantic category effects would also emerge during a task that required no explicit semantic categorization process, thus suggesting that brain activity related to semantic categories actually mirrored semantic memory organization. Thirdly, using the evoked potential technique, we attempted to examine how early a divergence between different semantic categories could be observed. We aimed to ascertain the possibility that ERP effects might be earlier than reported by previous studies, by taking under control some of the variables that play a crucial role in the early recognition of linguistic stimuli. Finally, source localization by means of LORETA was performed in an attempt to draw inferences about the specific cerebral regions involved in the first steps of automatic access to semantic information.

## Methods

### Participants

Sixteen undergraduate students took part in the experiment. They gave written informed consent to the experimental procedure, and they earned credits for participating. Four of them were excluded from statistical analysis because of excessive artifacts on EEG recording. The remaining twelve participants (6 men, 6 women) were all right-handed and aged between 20 and 25 years (Mean: 23; SD: 1.75). All had normal or corrected-to-normal vision and were in good health; none had ever suffered from neurological or psychiatric disorders. Handedness was assessed by the Italian version [25] of the Edinburgh Inventory Questionnaire [26]. Experiments were conducted in accordance with ethical standards (Helsinki, 1964).

### Stimuli and procedure

A total of 400 stimuli were selected. They comprised 100 Italian words, 100 legal derived pseudo-words, 100 non-derived pseudo-words, and 100 illegal letter strings. Derived pseudo-words were obtained by changing one single letter of an existing lemma (for example Colomba-Colemba), whereas non-derived pseudo-words were created ex novo and had no orthographic neighbors. Letter strings included both vocals (V) and consonants (C). The rationale and the analysis concerning the 4 lexical categories can be found in another paper [27]. Only behavioral and ERP responses associated with word stimuli were considered in the present paper.

Half the word stimuli were names of familiar animals (fauna); the remainder were names of familiar plants (flora). All stimuli were balanced in terms of length, which varied among 4 and 8 letters (fauna = 5.98; SD = 1.38; flora = 6.20; SD = 1.38). They were also balanced in terms of frequency of use (fauna = 21.53; SD = 37.11; flora = 22.82; SD = 29.33), familiarity (fauna = 3.92; SD = 0.91; flora = 4.05; SD = 0.76) and imageability (fauna = 3.97; SD = 0.58; flora = 4.15; SD = 0.50). A t-test was conducted for each of these parameters, and they were not significantly different up to the p =.1 level.

Word frequency was taken from a comprehensive online database of Italian words [28]. The frequency counts for the stimuli were measured in terms of absolute frequency. The reference corpus consists of excerpts from newspapers, magazines and books, including textbooks and books relating to professional interests, and comprises 3.798.275 lexical occurrences. Word familiarity and imageability were rated by sixty independent subjects who did not participate in the ERP experiment. The rating was based on a 5-point scale ranging from 1 to 5 using standardized instructions according to Snodgrass and Vanderwart [29]. For the familiarity rating scale, 5 = very familiar, 4 = fairly familiar, 3 = neither familiar nor unfamiliar, 2 = nearly unknown, and 1 = totally unknown. For the imageability rating scale, 5 = very hard to imagine, 4 = fairly hard to imagine, 3 = neither hard nor easy to imagine, 2 = fairly easy to imagine, and 1 = very easy to imagine.

Flora and fauna categories comprised a large number of stimuli, because our purpose was to have a broad base of individual concepts sharing their superordinate features. In order to balance the internal variability of the two categories, we chose exemplars belonging to four different subcategories. Among fauna names, there were four-footed animals, birds, insects, and fishes. Among flora names, there were fruits, vegetables, flowers, and trees. Moreover, we tried to select exemplars comparable for other aspects. For example, within each category there were names of things that can be eaten (fauna: salmon, chicken; flora: carrot, pineapple). Within each category there were exemplars very big or very small (fauna: ant, clam, elephant, whale; flora: blackberry, rose, oak, palm).

**Figure 1:**
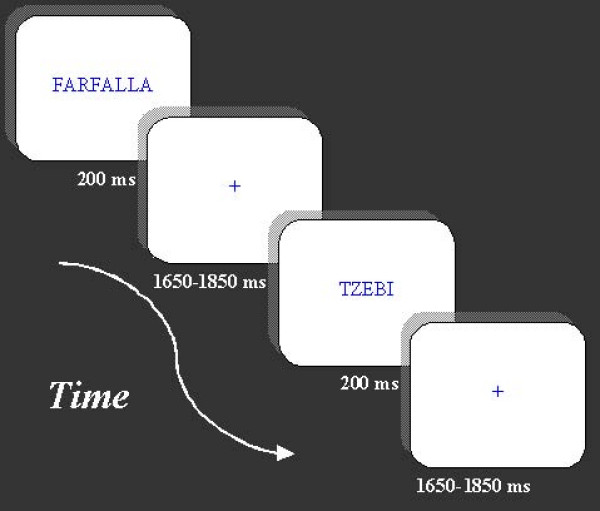
**Illustration of experimental procedure, with indication of stimulus duration (in ms) and inter-stimulus interval**.

The stimuli were presented at the center of a PC monitor. They were typed in Times New Roman capital letters, and they were blue on a white background (see Figure 1). Their lengths ranged from 4 to 9 cm. They were 1 cm in height and subtended visual angles of 0°30'10" on the vertical axis and 2°1'41" to 4°32'32" on the horizontal axis.

Six blocks of trials were created. Five of them contained 68 stimuli, the remained contained 60 stimuli. The repartition per block was done so that the same number of trials belonging to each category (words, derived pseudo-words, non derived pseudo-words, strings) was presented within each block. For example, block one contain 17 words (about half flora names, half fauna names), 17 derived pseudo-words (half derived from flora names, half derived from fauna names), 17 non derived pseudo-words, and 17 strings randomly distributed. Each block lasted about 2.5 minutes and was preceded by three warning signals ("ready", "steady", "go"), presented for 500 ms. Each stimulus remained on the screen for 200 ms and was followed by a 1650–1850 ms random ISI. The order of presentation of the blocks was counterbalanced across subjects.

The participants were seated in an acoustically and electrically shielded box 114 cm from the screen. They were instructed to fixate a cross in the centre of the screen and minimize any eye or body movement during the recording period.

The task consisted of deciding as quickly and accurately as possible whether or not the stimulus was a real word (lexical decision task). Responses were made by pressing one of two buttons with the index finger (if the stimulus was a real word) or the middle finger (if the stimulus was a non-word). The two hands were used alternately during the recording session. The order of hand use was counterbalanced across subjects. Before the experimental session, the participants were given written and oral instructions about the task, and they were presented with two blocks of training trials similar to the experimental trials. See Table 1 for a list of example stimuli.

**Table 1:** Examples of words belonging to the two lexical classes.

**Fauna (Italian)**	***English***	**Flora (Italian)**	***English***
allodola	*lark*	ananas	*pineapple*

anatra	*duck*	bietola	*beet*

aquila	*eagle*	dattero	*dates*

asino	*donkey*	edera	*ivy*

cane	*dog*	fico	*fig*

capra	*goat*	fragola	*strawberry*

corvo	*crow*	fungo	*mushroom*

criceto	*hamster*	girasole	*sunflower*

delfino	*dolphin*	magnolia	*magnolia*

elefante	*elephant*	ortica	*nettle*

farfalla	*butterfly*	palma	*palm*

foca	*seal*	pigna	*cone*

gallo	*cock*	primula	*primrose*

rondine	*swallow*	quercia	*oak*

rospo	*toad*	salice	*willow*

### EEG recording and analysis

The electroencephalogram (EEG) was continuously recorded from 128 scalp sites using tin electrodes mounted in an elastic cap (Electro-Cap) and according to the international 10-5 system [30], at a sampling rate of 512 Hz.

In order to monitor blinks and vertical eye movements, two electrodes were placed below and above the right eye (vEOG channel), while horizontal movements were monitored by means of two electrodes placed at the outer canthi of the eyes (hEOG channel). Averaged ears served as the reference lead. The EEG was recorded using Cognitrace software (ANT Software, Enschede, The Netherlands), and was amplified using an ANT amplifier, with a half-amplitude band pass of 0.016–100 Hz. Electrode impedance was kept below 5 kΩ. The EEG was analyzed using EEProbe software (ANT Software, Enschede, The Netherlands). Computerized artifact rejection was performed before averaging to discard epochs in which eye movements, blinks or excessive muscle potentials occurred. The artifact rejection criterion was a peak-to-valley amplitude exceeding ± 50 μV. The baseline was corrected from 100 ms before to the onset of the stimulus. ERPs were averaged offline from 100 ms before to 1000 after presentation of the stimulus. EEG epochs were synchronized with stimulus onset and ERP trials associated with an incorrect behavioral response were excluded from further analysis. After the off-line averaging, ERPs were treated with a 40 Hz low-pass filter.

Topographical voltage maps of ERPs were made by plotting color-coded isopotentials obtained by interpolating voltage values between electrodes at specific latencies. Low Resolution Electromagnetic Tomography or LORETA [31] was performed on ERP difference waves at specific latencies using ASA4 software (ANT Software, Enschede, The Netherlands). LORETA is a discrete linear solution to the inverse EEG problem, and corresponds to the 3D distribution of neuronal electric activity that has maximum similarity (i.e. maximum synchronization), in terms of orientation and strength, between neighboring neuronal populations (represented by adjacent voxels). In this study, an improved version of Standardized LORETA (swLORETA) was used that incorporates a singular value decomposition-based lead field weighting [32]. Source space properties were: grid spacing = 5 mm; Tikhonov regularization: estimated SNR = 3.

For each participant, reaction times exceeding mean ± 2 standard deviations were excluded. Mean reaction times, arcsin-transformed percentages of errors, and mean amplitudes of the major ERP components were subjected to multifactorial repeated-measures ANOVA. Factors included "semantic category" (flora, fauna) and "response hand" (left, right) for behavioral data; and "semantic category" (flora, fauna), "electrode", (dependent on the ERP component of interest) and "hemisphere" (left, right) for electrophysiological data. In particular, occipito-temporal N2 was measured at lateral occipito-temporal sites (P7, P8, TPP9h, TPP10h) between 170 and 220 ms; centro-parietal N400 was measured at central (C3, C4) and centro-parietal (CP5, CP6) sites between 320 and 470 ms; posterior P300 was measured at centro-parietal (CP1, CP2) and occipito-lateral (PO9, PO10) sites between 470 and 620 ms; and anterior LP was measured at fronto-central sites (FPz, AFz) between 530 and 680 ms. Post-hoc Tukey tests were used for multiple comparisons among means.

## Results

### Behavioral results

RTs tended to be faster in responding to words denoting animals (fauna) than to words denoting plants (flora) (Fauna = 551 ms, SD = 48.32; Flora = 562 ms, SD = 53.70). Nevertheless ANOVA performed on response times to fauna and flora names indicated no significant effects (F1,11 = 2.88; p = 0.12).

Analysis of accuracy (error percentages) showed an effect of semantic category (F1,11 = 6.42; p < 0.05; eta2 = 0.37; F-crit = 4.84): subjects were more accurate in responding to fauna than to flora names (Fauna = 4.89%; Flora = 8.85%).

### Electrophysiological results

**Figure 2:**
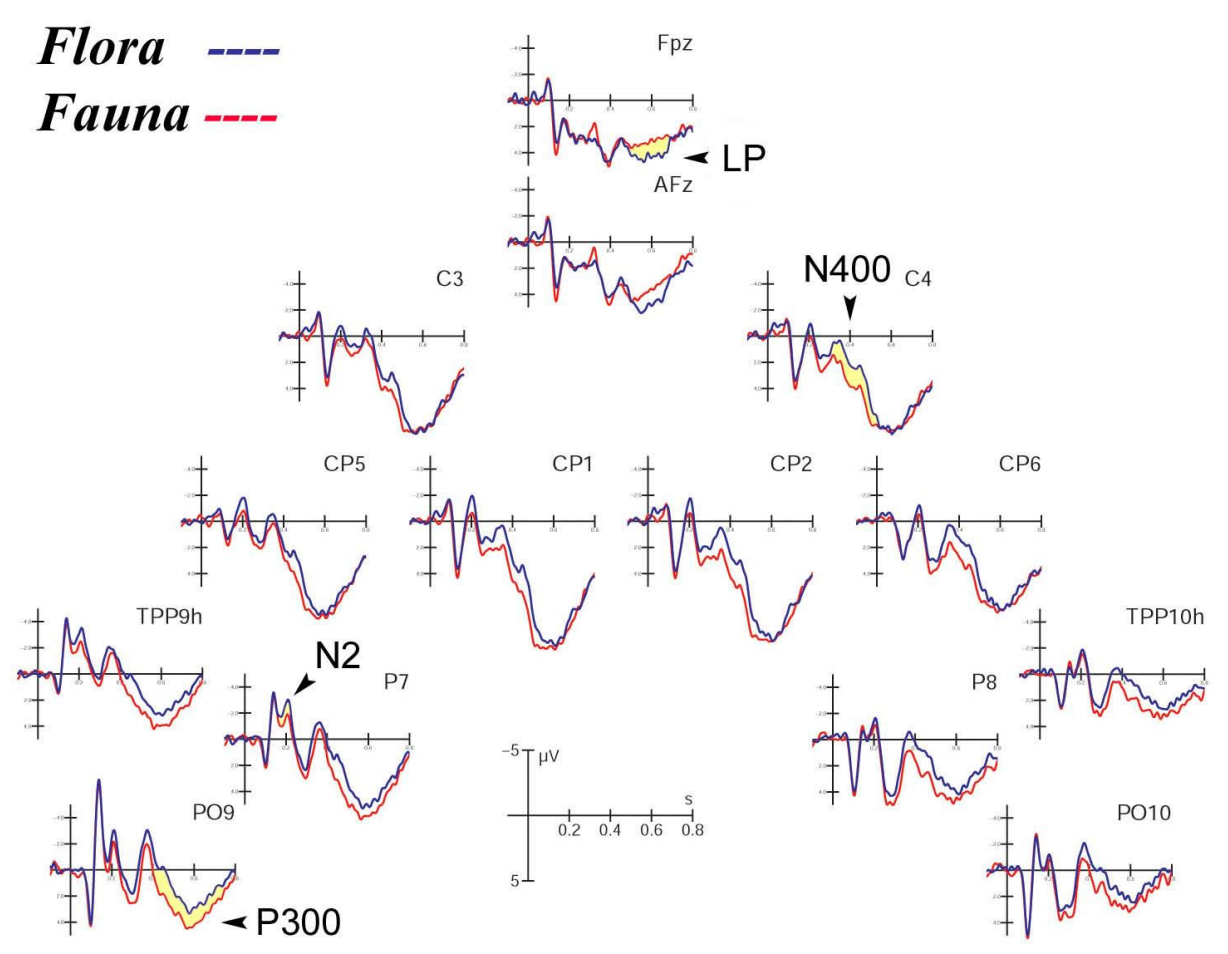
**Grand-average ERP waveforms recorded at fronto-central, centro-parietal, occipito-temporal and occipito-lateral sites in response to flora and fauna**. The arrows indicate the ERP components considered in this study.

Figure 2 shows the grand-average ERP waveforms recorded at the fronto-central, centro-parietal, occipito-temporal and occipito-lateral sites in response to the two stimulus types and represents a summary of category effects for the amplitudes of all components described below.

The analysis of P1 component, which reached its maximum amplitude at occipital sites at about 100, revealed no significant effects. Similarly, the analysis of N1 component, which reached its maximum amplitude at occipito-lateral sites at about 140 ms, revealed that the semantic category of the stimulus did not significantly modulate the amplitude of this component.

#### Occipito-temporal N2 component (170–220 ms)

**Figure 3:**
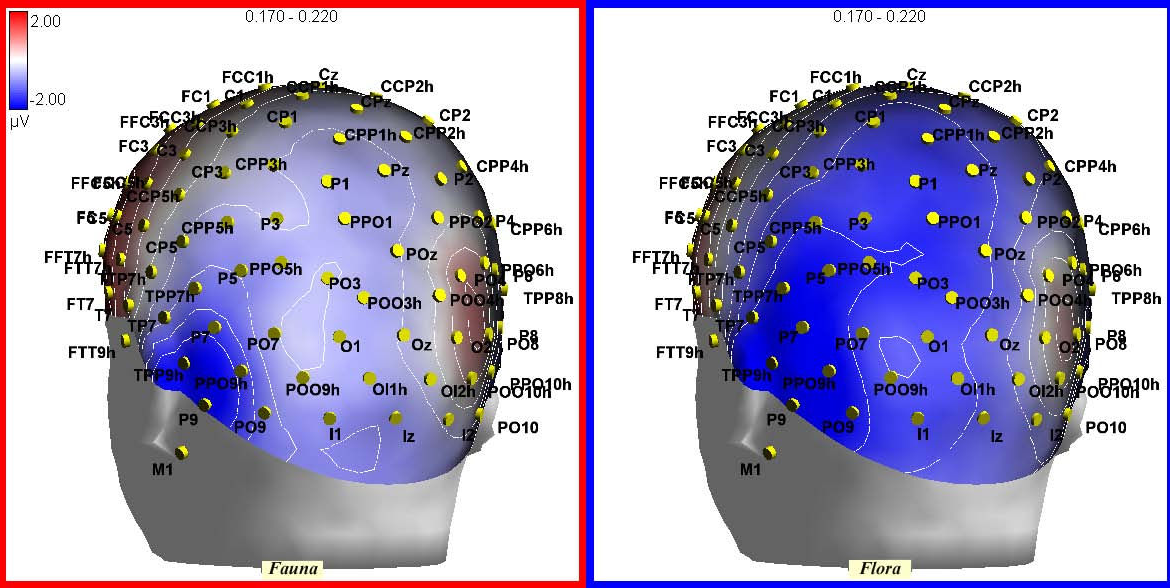
**Back view of topographical distribution of voltage obtained in response to fauna names and in response to flora names during the 170–220 ms post-stimulus interval**.

This component reached its maximum amplitude at the lateral occipito-temporal sites (P7, P8, TPP9h, TPP10h). N2 was strongly affected by semantic category, as suggested by the "Semantic category × Hemisphere" interaction (F1,11 = 5.93; p < 0.05; eta2 = 0.35; F-crit = 4.84), showing larger amplitudes in response to words denoting flora than fauna over the left but not the right hemisphere (LH: Fauna = -1.69; Flora = -2.63 μV; RH: Fauna = -0.78; Flora = -1.02 μV, see Figure 3).

**Figure 4:**
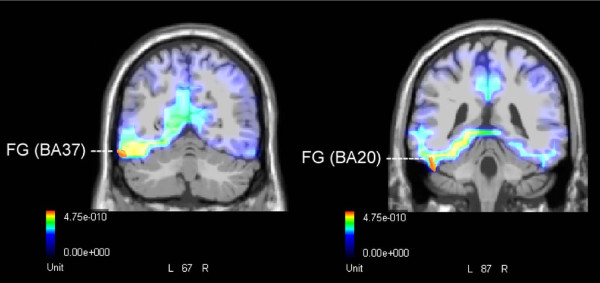
**Source localization relative to difference voltage obtained by subtracting grand-average ERPs to fauna names from ERPs to flora names (**flora – fauna**) during the 170–220 ms post-stimulus interval**. The solution showed a strong source of activation located in the left inferior temporal gyrus (BA37 and BA20). Grid spacing = 5 mm; Tikhonov regularization: estimated SNR = 3; Power RMS = 17.8 μV.

LORETA source reconstruction was performed on the difference wave obtained by subtracting the ERP responses to words denoting fauna from those denoting flora in the occipito-temporal N2 latency range (170–220 ms). As indicated in Table 2 and Figure 4, the solution showed a strong focus of activity in the left inferior temporal lobe, in particular in BA37 and BA20.

**Table 2:** Talairach coordinates corresponding to intracranial generators explaining the distribution of voltages recorded in response to "flora minus fauna" names between 170 and 220 ms.

**Magnitude** **(E-10)**	**T-x** **[mm]**	**T-y** **[mm]**	**T-z** **[mm]**	**Hemisph**.	**Lobe**	**Gyrus**	**BA**
4.75	-29	-1	-28	L	Limbic	Uncus	36

4.43	-49	-34	-24	L	T	Fusiform Gyrus	20

4.38	-59	-55	-18	L	T	Fusiform Gyrus	37

4.37	51	34	14	R	F	Middle Frontal Gyrus	46

3.93	11	-9	-14	R	Limbic	Parahippocampal Gyrus	34

3.88	21	9	-28	R	Limbic	Uncus	38

3.85	-9	57	-9	L	F	Superior Frontal Gyrus	10

3.83	-29	53	25	L	F	Superior Frontal Gyrus	10

3.62	-29	56	-2	L	F	Superior Frontal Gyrus	10

3.51	2	-29	26	R	Limbic	Cingulate Gyrus	23

3.47	51	-1	-28	R	T	Middle Temporal Gyrus	21

3.28	51	-34	-24	R	T	Fusiform Gyrus	20

2.36	41	-30	35	R	P	Inferior Parietal Lobule	40

2.31	31	-52	42	R	P	Superior Parietal Lobule	7

#### Centro-parietal N400 component (320–470 ms)

The N400 component reached its maximum amplitude at the central (C3, C4) and centro-parietal (CP5, CP6) scalp sites. At this scalp region the effect of semantic category (F1,11 = 7.37; p < 0.05; eta2 = 0.40; F-crit = 4.84) indicated a greater N400 to flora than fauna names (Fauna = 2.80; Flora = 1.58 μV).

#### Posterior P300 component (470–620 ms)

The posterior P300 component was larger at the centro-parietal than the occipito-lateral area (CP1-CP2 = 8.46; PO9-PO10 = 2.55 μV), as proved by the electrode factor (F1,11 = 49.90; p < 0.001; eta2 = 0.82; F-crit = 4.84). It was strongly affected by semantic category (F1,11 = 11.38; p < 0.01; eta2 = 0.51; F-crit = 4.84): fauna names elicited larger P3 responses than flora names (Fauna = 6.04; Flora = 4.97 μV).

Post-hoc comparisons for the triple interaction "Semantic category × Electrode × Hemisphere" (F1,11 = 6.90; p < 0.05; eta2 = 0.39; F-crit = 4.84) confirmed that fauna names elicited larger P3 responses than flora names at all electrode sites (p < 0.001; PO9: Fauna = 3.72; Flora = 2.32 μV; PO10: Fauna = 2.57; Flora = 1.61 μV; CP1: Fauna = 9.23; Flora = 8.23 μV; CP2: Fauna = 8.65; Flora = 7.72 μV).

**Figure 5:**
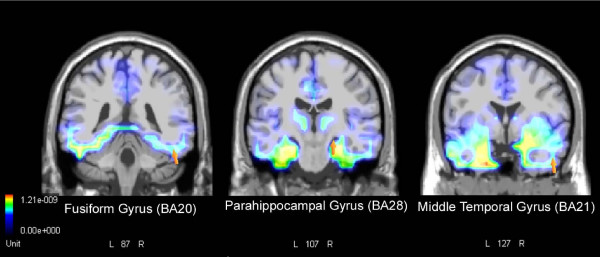
**Source localization relative to difference voltage obtained by subtracting grand-average ERPs to flora names from ERPs to fauna names (**fauna – flora**) during the 470–530 ms post-stimulus interval**. The solution showed strong activation of the occipito-temporal regions of the right hemisphere. The Figure shows dipoles located in the fusiform gyrus (BA20), parahippocampal gyrus (BA28) and middle temporal gyrus (BA21). Grid spacing = 5 mm; Tikhonov regularization: estimated SNR = 3; Power RMS = 34.1 μV.

LORETA source reconstruction was performed on the difference wave obtained by subtracting the ERPs to words denoting flora from those elicited by fauna names in the peak latency (470–530 ms). As reported in Table 3, the solution showed strong activation of the occipito-temporal regions of the right hemisphere (Figure 5), in particular the inferior occipital gyrus (BA18), middle occipital gyrus (BA19), fusiform gyrus (BA20), parahippocampal gyrus (BA28) and middle temporal gyrus (BA21), as well as a strong focus of activity in the left inferior temporal lobe (BA20).

**Table 3:** Talairach coordinates corresponding to intracranial generators explaining the distribution of voltages recorded in response to "fauna minus flora" names between 470 and 530 ms.

**Magnitude** **(E-10)**	**T-x** **[mm]**	**T-y** **[mm]**	**T-z** **[mm]**	**Hemisph**.	**Lobe**	**Gyrus**	**BA**
12.1	-19	-8	-29	L	Limbic	Uncus	36

11.7	-59	-45	-17	L	T	Inferior Temporal Gyrus	20

11.1	21	-17	-15	R	Limbic	Parahippocampal Gyrus	28

9.59	51	-34	-24	R	T	Fusiform Gyrus	20

9.35	51	-1	-28	R	T	Middle Temporal Gyrus	21

7.68	2	-20	27	R	Limbic	Cingulate Gyrus	23

6.58	-49	45	6	L	F	Middle Frontal Gyrus	46

6.37	-29	56	-2	L	F	Superior Frontal Gyrus	10

6.17	41	33	23	R	F	Middle Frontal Gyrus	46

5.78	2	65	8	R	F	Medial Frontal Gyrus	10

3.90	31	-98	-6	R	O	Inferior Occipital Gyrus	18

2.87	41	-30	35	R	P	Inferior Parietal Lobule	40

#### Anterior LP component (530–680 ms)

The anterior LP had a fronto-central (FPz, AFz) distribution. In contrast to posterior P300, larger positivities in response to words denoting flora than fauna were observed at the fronto-central sites (Fauna = 3.33; Flora = 4.47 μV), as suggested by the semantic category effect (F1,11 = 4.86; p < 0.05; eta2 = 0.31; F-crit = 4.84).

## Discussion

### Early ERP effects

The present study confirms the existence of an early ERP effect (170–220 ms) of semantic category: the occipito-temporal N2 component was characterized by a larger amplitude in response to words denoting flora than fauna over the left but not the right hemisphere. The strict left lateralization of this effect suggests the linguistic nature of the phenomenon. In contrast to previous studies [18,20], we demonstrated that the effect found in this time window was due neither to word imageability nor to concept familiarity, as these two factors were balanced. What actually sets the two semantic categories apart remains to be clarified, considering that the task used did not require or benefit from the analysis of a particular property; and, as mentioned above, imageability as well as familiarity and written frequency of occurrence of the two semantic categories were balanced. Generally speaking, fauna names could be discriminated from flora names, at least in part, because of biological relevance, more significant in the case of fauna names. In other words, the homomorphic properties of fauna (that is, the presence of eyes, head, etc.) could make them "unique" compared to flora names, which although often assimilated under "living entities" in the semantic category literature, do not possess such features and can therefore be placed "halfway" between living and non-living things. This hypothesis is consistent with the assumption of the Conceptual-Structure account [6] that biological function information would be highly correlated to the shared perceptual properties of living entities (that is, the presence of eyes, head, etc.), whereas functional information would be highly correlated with the distinctive perceptual properties of non-living things (for example, it's used for spearing/it has tines).

It is worth noting that some researchers tried to identify the variables that might be relevant to the organization of semantic memory by taking a different approach. In the so-called norming task, subjects were given a sequence of concept names and they were asked to list features of the things to which the words refer. McRae and Cree concluded that perceptual and semantic feature correlations seemed to be more plentiful and strong within living things with respect with non living things, in particular within the animal category [33]. In the light of this, it seems reasonable to hypothesize that the shared perceptual properties of fauna might in part contribute to the emerging differentiation between flora and fauna lexico-semantic categories.

The topographical voltage distribution of ERPs and LORETA source reconstruction suggest a strong involvement of the extrastriate visual areas of the ventral pathway in lexical processing, in particular of the left fusiform gyrus (BA37 and BA20). The involvement of these areas in lexical processing is well documented in neuroimaging literature. Indeed, many neuroimaging studies have shown that the left fusiform cortex responds with greater activation to linguistic than non-linguistic stimuli. This area seems to play a crucial role in visual word form representation, and it is often called the Visual Word Form Area or VWFA [34,35]. Recent ERP-LORETA studies of the present research group seem to provide strong support to this hypothesis. For example, in a recent study, Proverbio and collaborators [36] recorded event-related potentials to words in standard vs. mirror orientation during a letter detection task. Word inversion was performed to determine whether rotated words lose their linguistic properties. N1 (135–215 ms) component was greater to rotated than standard words, and to target than non-target letters at the left lateral occipital sites. LORETA source reconstruction revealed a strong focus of activation for the effect of target letter selection in the left fusiform gyrus (BA37). In another recent study, Proverbio and collaborators [37] found larger N2 and N3 components to high-frequency than low-frequency words or pseudo-words within the left lateral occipital areas. The solution provided by LORETA indicated greater left fusiform (BA19) and right superior temporal (BA22) activation for processing high frequency than low frequency words. In a final study, aimed at investigating the role of orthographic familiarity, phonological legality and number of orthographic neighbors of words in determining the onset of word/non-word discriminative responses, the findings of Proverbio and co-workers [27] confirm that the left occipito-temporal area, in particular BA37, was sensitive to word visual familiarity. In the present study, LORETA source reconstruction showed a strong focus of activity in more anterior regions, namely BA20, besides activation of BA37. The activation of BA20 might be linked to lexico-semantic access, a "higher level" linguistic processing as respect to the orthographic processing described in the previous papers. However, it must be kept in mind that as a method for localizing the electric activity in the brain based on surface EEG recordings, LORETA is characterized by a relatively low spatial resolution. Then, our results should be evaluated by their convergence with neuroimaging and lesion data. Interestingly, our finding goes in the same direction as the MEG data of Assadollahi and Rockstroh [21] previously mentioned. These authors found an effect of flora names vs. fauna names as early as 100–150 ms in the left occipito-temporal cortex. This region is more anterior than the region reported by Assadollahi and Pulvermüller [15] for the effects of word frequency and length. Assadollahi and Rockstroh interpreted this effect in terms of semantic rather than orthographic processing. It is worth noting that some authors identified an area located anterior to the VWFA, along the anterior fusiform gyrus, sensitive to semantic manipulations, both for words and for object pictures [13].

### Later effects

Overall, analysis of the later ERP components and behavioral data both suggest that flora names somehow required more effortful cognitive resources than fauna names. Subjects were significantly less accurate in responding to flora than fauna names, and mean RTs to fauna names were a little bit faster than to flora names, even though analysis of RTs indicated no significant effect. Similarly, Kiefer [8], using a lexical decision task on words denoting natural entities (animals, plants, fruits, and vegetables) or artifactual things (tools, furniture, transportation, and musical instruments), found a marginally significant main effect of category, showing that reactions to natural categories were slightly faster than to artifactual categories.

The analysis of the centro-parietal N400 showed a greater amplitude to flora than fauna names. According to the literature, the N400 component might represent an index of the difficulty with which input information is integrated with previously stored semantic information [38]. Some authors have suggested that N400 could reflect a sort of research into the long-term semantic memory. Indeed, its amplitude varies according to factors that also affect long term memory, such as the number of items to be recalled [39] and the delay times among different presentations of a certain item [40]. In the light of the N2 data, it is also possible that decision-making might have been more difficult for flora names, which are less homogeneous than fauna names. Indeed, fauna names are characterized by common visual properties (all animals have eyes, a head), as well as common semantic properties (they all are living entities that are able to move, eat, breathe etc.). The activation of intercorrelated and shared features might have facilitated automatic recognition processing.

The anterior LP component, like N400, showed larger positivities in response to words denoting flora than fauna. In the previous literature, ventro-lateral prefrontal activity has been strongly associated with top-down control of semantic memory [41], particularly the selection of conceptual information stored in the posterior temporal cortex (and presumably in other cortices) during retrieval processing [42]. Although the specific role of the ventro-lateral prefrontal cortex still remains a matter of debate, there is some agreement that its main function is to control and modulate access to information stored elsewhere [43]. Again, this component could prove that the processing of flora names was a more demanding task.

Fauna names elicited larger occipito-parietal P3 responses than flora names. This may indicate more sensory associations of fauna names within the extra-striate visual areas. This hypothesis is supported by LORETA source reconstruction, which suggests a large involvement of the right occipito-temporal regions, in particular the inferior occipital gyrus (BA18), middle occipital gyrus (BA19), fusiform gyrus (BA20), parahippocampal gyrus (BA28) and middle temporal gyrus (BA21), as well as a strong focus of activity in the left inferior temporal lobe (BA20). Our finding is compatible with neuroimaging studies showing activation of the ventral occipital and the temporal cortex during processing of names of living entities versus other concepts. The involvement of visual areas has often been interpreted as associated with the sensory richness of the visual features of living entities [44,45]. For instance, in a recent fMRI study, Marques and co-workers [46] found strong activation of the inferior temporal and fusiform gyri in the temporal lobe and a wide occipito-parietal region extending from the dorsal middle occipital gyrus to the inferior parietal lobule according to the retrieval of visual form/surface features of different concepts. These regions were located in both hemispheres but with a right hemispheric prevalence. Given that fauna and flora names were balanced in terms of imageability in the present study, the greater activation of visual extrastriate areas does not seem to be attributable simply to different degrees of imageability of the two categories. It is relevant to note that activation of the right occipito-temporal cortex has often been associated with face and body processing, the so-called Face Fusiform Area and Extra-striate Body Area [47,48]. Again, this result seems to suggest that the effects found are related to biological function information, highly correlated to shared perceptual and semantic properties.

With regard to the theoretical accounts mentioned in the introduction, the finding of an early dissociation between fauna and flora names seems incompatible with the Sensory/Functional Theory [1,2], which on the basis of its assumptions predicts that no dissociations should be observed within the living things category. We found a clear distinction between the two semantic categories in term of ERP amplitude, but we failed to find differences in the topographical distributions of the same components. Thus, our results are in part compatible with a model in which neural circuits responsible for processing different object-categories exist. Nevertheless, our findings suggest that the lexical processing of flora and fauna names used, balanced in terms of a number of linguistic properties (and quite close in terms of semantic features as respect to the previous literature), were not characterized by a strong neuro-functional specialization [5]. However it can not completely ruled out, considering the relatively poor spatial resolution of EEG technique.

Anyway, the localization of functional activity per se is not sufficient to come down in favor of the hypothesis that different category-related representations do exist. The hypothesis that perceptual features play a crucial role in comprehending the names of living things while functional/associative features play a crucial role in comprehending the names of non-living things [2] can not be excluded. More direct tests of the opposing theories are needed. A number of studies have manipulated both domain (living vs. non-living) and feature type [49,50]. In a recent fMRI study, Marques and his collaborators [46] evaluated the role of domain (living vs. non-living) and feature type (visual form/surface vs. motion) through a concept-feature verification task. Each item was embedded in a simple sentence (e.g. Snake is long – living concept/visual feature; Water hose is long – non living concept/visual feature; Snake rolls up – living concept/motion feature; Water hose rolls up – non living concept/motion feature), and participants were requested to decide whether the statement presented was true or false. The retrieval of visual form/surface features, as opposed to motion features (independent of concept domain), activated areas involved in high-order visuo-perceptual processing, such as the inferior temporal and the medial fusiform gyrus (BA37), and a wide occipito-parietal region extending from the dorsal middle occipital gyrus to the inferior parietal lobule, predominantly in the right hemisphere. There was no specific effect of semantic domain (living vs. non-living item) on brain activation. The authors concluded that their findings were in accord with a modality-specific account of conceptual knowledge organization in the brain.

To summarize, the effects found in our study are fairly consistent with the hypothesis that living entities, in particular animals, have many shared properties that co-occur frequently and are strongly related to biological function information (Conceptual-Structure account), or in other words, with the idea that fauna names and the underlying concepts are highly correlated to shared perceptual and semantic features [6]. This may guide and facilitate automatic lexico-semantic recognition processing. In any case, we believe that despite their limitations, each account mentioned provides insights into important aspects of semantic processing, and an account that integrates the properties of each will be most successful. Regarding the Conceptual-Structure account, the same authors recognized that the assumption that conceptual information is randomly distributed with no category/domain organization is likely to be an oversimplification. Some authors have proposed alternative solutions, suggesting that both the domain and feature type (such as orthogonal dimensions) would subsume the organization of conceptual knowledge [51,52].

### A possible role for Age of Acquisition?

For future research it is important to consider that, beside perceptual and functional properties of the two different categories, the word Age of Acquisition might importantly affect the lexico-semantic processing of words belonging to different categories. At present, it was not possible to balance the Age of Acquisition (AoA) of words because there are no Italian norms that would include all the stimuli used in this study. Models have been proposed suggesting that words learned early and late are represented differently in the brain [53]. However, it is currently unclear whether Age of Acquisition or word frequency are better predictors of word recognition [54], and few studies have investigated the neural bases of word Age of Acquisition. For instance, Fiebach and collaborators [55] reported a visual and auditory fMRI experiment investigating the influence of Age of Acquisition and word frequency on neural activity, and found that Age of Acquisition modulates brain areas that are not influenced by word frequency. The precuneus was activated for early-learned words across auditory and visual presentation modalities. Late-learned words, in contrast, led to a selective increase of activation in the lateral inferior frontal areas. The authors hypothesized that early-learned words are represented in the brain in a more sensory manner than late-learned words. In an ERP study involving an auditory lexical decision task, Tainturier and collaborators [56] found larger P300 in response to early-acquired words than late-acquired words, other factors (length, concreteness, imageability and word frequency) being balanced. Interestingly, the results of P300 component of the present experiment goes in the same direction as the effect described by these authors, and is thereby consistent with the hypothesis that Age of Acquisition played a significant role in our experiment.

The importance of Age of Acquisition in word processing has been suggested also in the multilingualism literature. For instance, in a recent study investigating the timing of brain activation during processing of native vs. later-acquired languages in simultaneous interpreters, Proverbio and co-workers [57] reported early lexical effects (words vs. pseudo-words) at occipito-temporal sites at about 160–180 ms only for the native and not the second language, although the interpreters were equally proficient in both languages and the task (letter detection) did not require lexico-semantic processing.

With regard to our results, an interesting possibility arises from the normative data of Caselli and co-workers [58] about the Italian version of the "MacArthur-Bates Communicative Development Inventory-CDI". This questionnaire, administered to parents of infants aged 18–36 months, is used to investigate and evaluate language and communication in normal and atypical development during the first years of life. Parents are asked to check the words that their child says spontaneously; the total number of checked words gives an estimate of overall productive vocabulary size. Normative data refer to the percentage of infants (in a sample group of 752) who, as reported by their parents, produced a certain word. Words are subdivided into various subclasses, such as animals, food, vehicles, etc. Interestingly, 17 of the fauna names used in the present study appear in this dataset compared to eight flora names. Moreover, a specific section in the questionnaire is dedicated to fauna names, whereas flora names are dispersed in the section dedicated to "food and drinks" and "outdoors". It is possible that concept acquisition and classification of members of the two categories take place in different ways and have distinguishing cognitive and affective features, considering that fauna seems to be conceptualized from an early age as a group of entities pooled by their biological relevance, whereas flora could be conceptualized later, in cognitive terms, as represented by all entities belonging to a scientifically defined kingdom. In this sense, our considerations about the word Age of Acquisition coincide with the foregoing discussion about the homomorphic features of fauna. Instead of a single word Age of Acquisition, which probably did not differ much among fauna and flora names, since the two categories were balanced in terms of frequency of occurrence, concept familiarity, and they all were names of concrete high imaginable concepts, our considerations about the Age of Acquisition should be seen in the context of more general knowledge about the two domains. In our culture it is possible that members of the two categories are acquired during different life periods and contexts, and this could be due in part to the biological/affective relevance of different concepts. In our case, the fact that fauna might be acquired in more "natural" contexts and earlier in life than flora might have resulted in a greater readiness to process the former category. It is possible that a later and/or a less consistent acquisition of concepts referring to the flora domain resulted in the enhanced N2, N400, and LP components, given that these effects are unlikely to be due to other controlled factors, like general familiarity with concepts during adulthood (familiarity having been balanced), word imageability or word frequency of occurrence.

Of course, this study was not specifically designed to look at Age of Acquisition, and the discussion of this issue is highly speculative. However, in our opinion further studies are needed to clarify the role of this important factor on semantic processing of word stimuli.

## Conclusion

In summary, our results indicate the existence of semantic category effects at early latencies and in brain regions usually involved in semantic category processing [51]. This effect seems not to be strictly bound to word imageability or familiarity. Implicit semantic processing occurred even if it was not required by the task. Our results also indicate that semantic processing may take place at a surprisingly early stage and near-simultaneously with the processing of information about the form of a word and its lexical properties.

With respect to the semantic category-related effects found in the present study, we would like to emphasize that in the context of our culture it is possible that the members of the two categories considered here are acquired during different life periods and contexts. This aspect could be due in part to the biological/affective relevance of different concepts. In our opinion, further studies are needed to clarify the role of general factors such as the age at which words/concepts are acquired, apart from the importance of specific features of concepts such as the number of clusters of intercorrelated features, modality, and domain in lexico-semantic brain processing.

## List of abbreviations

ANOVA: analysis of variance; RTs: reaction times; EEG: electroencephalogram; EOG: electro-oculogram; ERPs: event-related potentials; LORETA: low resolution electromagnetic tomography; BA: Brodmann area; MEG: magnetoencephalography.

## Competing interests

The authors declare that they have no competing interests.

## Authors' contributions

AMP conceived of the study, coordinated data acquisition and analysis, interpreted the data and worked on the manuscript. RA participated in the design of the study, collected the data, performed statistical analyses and source localization, drafted the manuscript. All authors read and approved the final manuscript.
